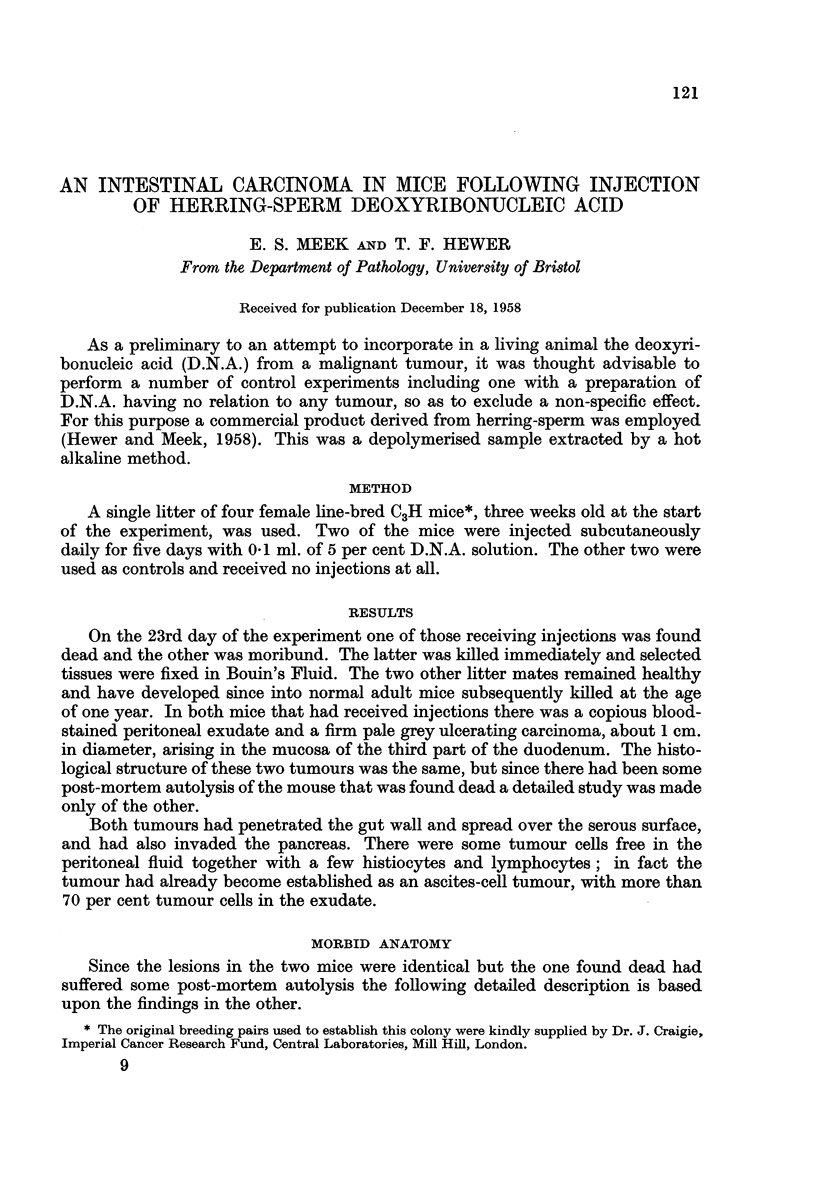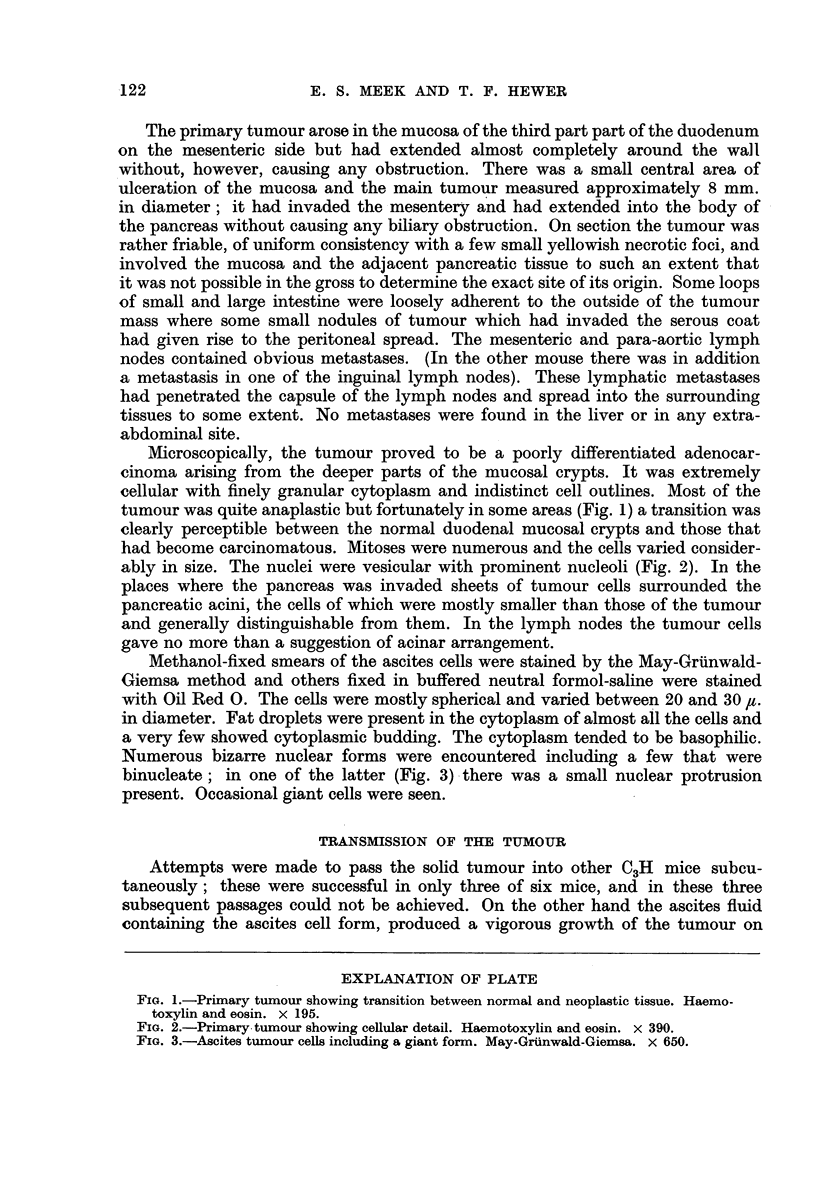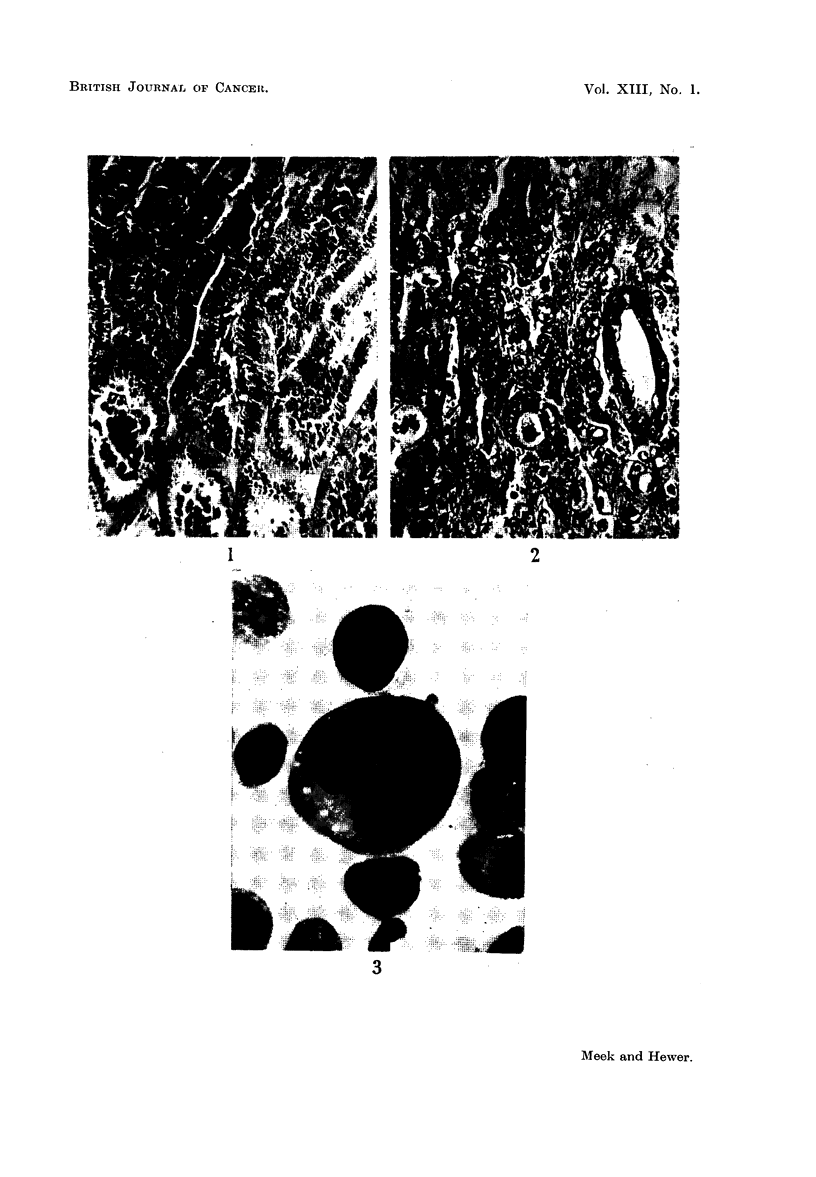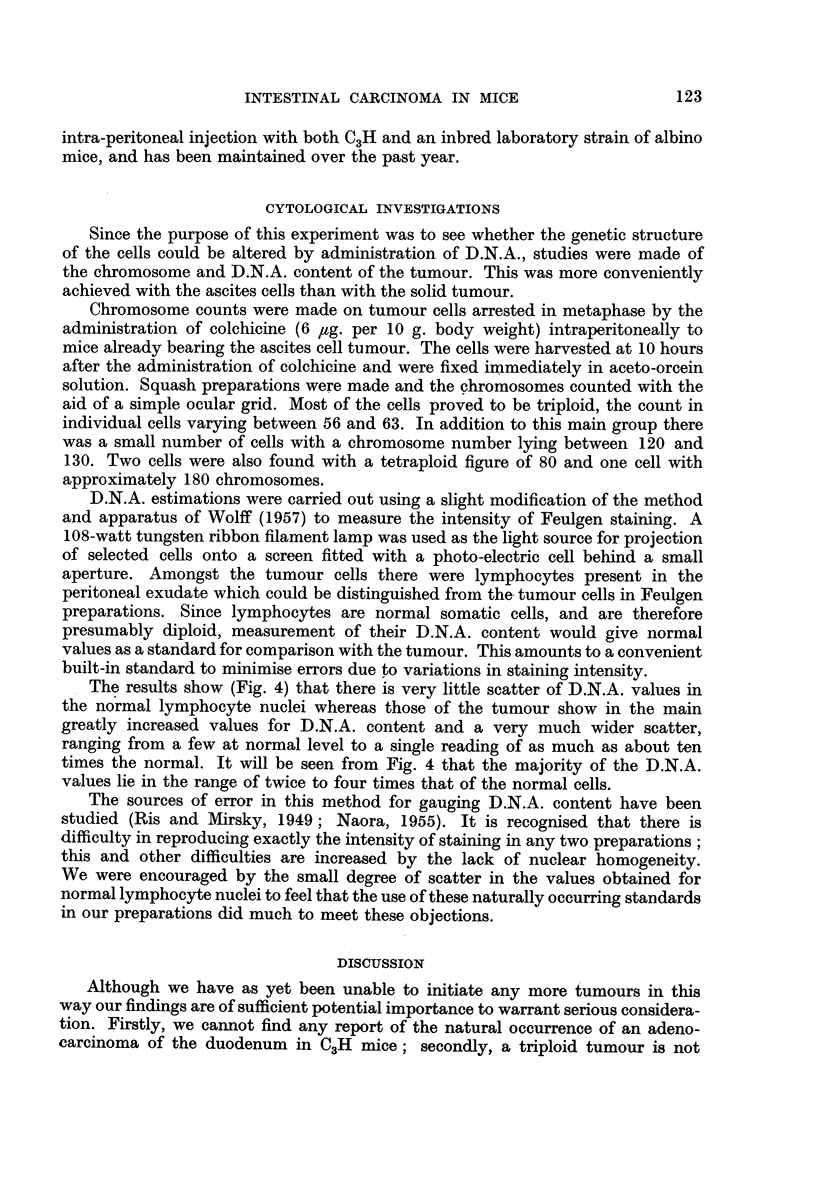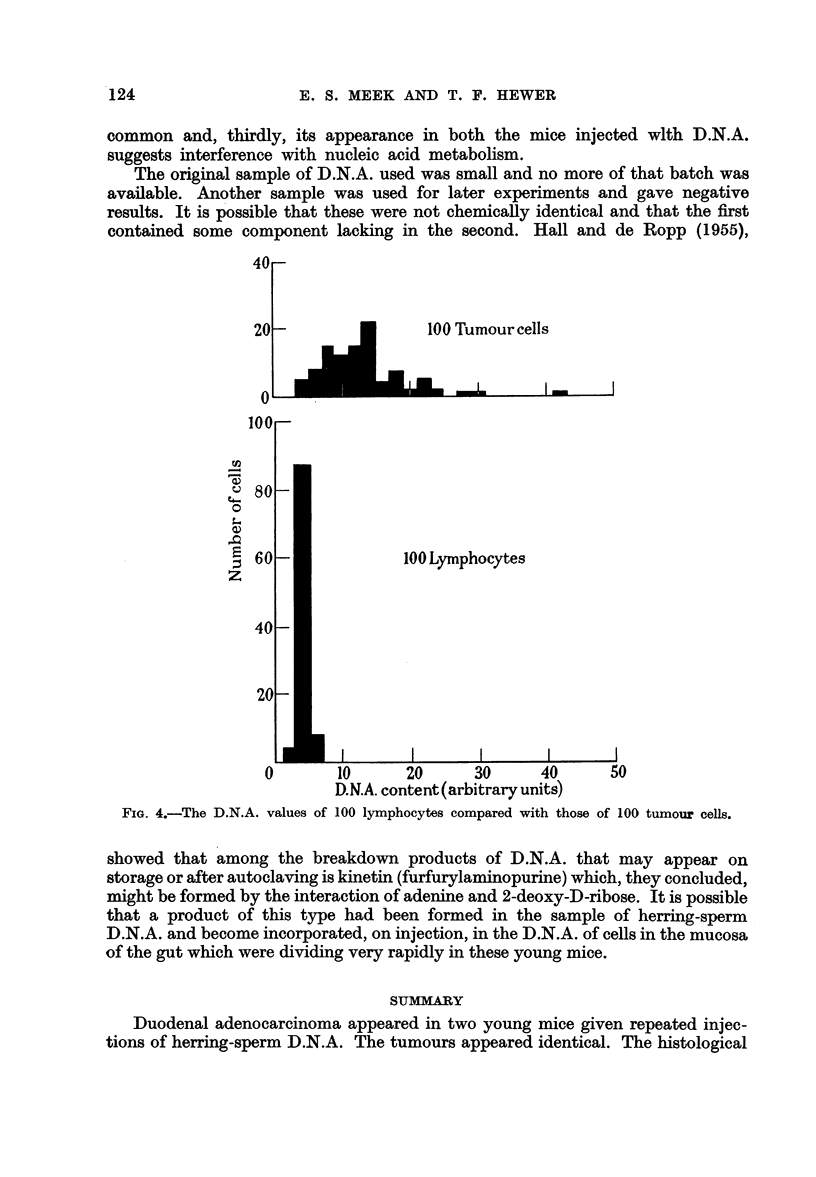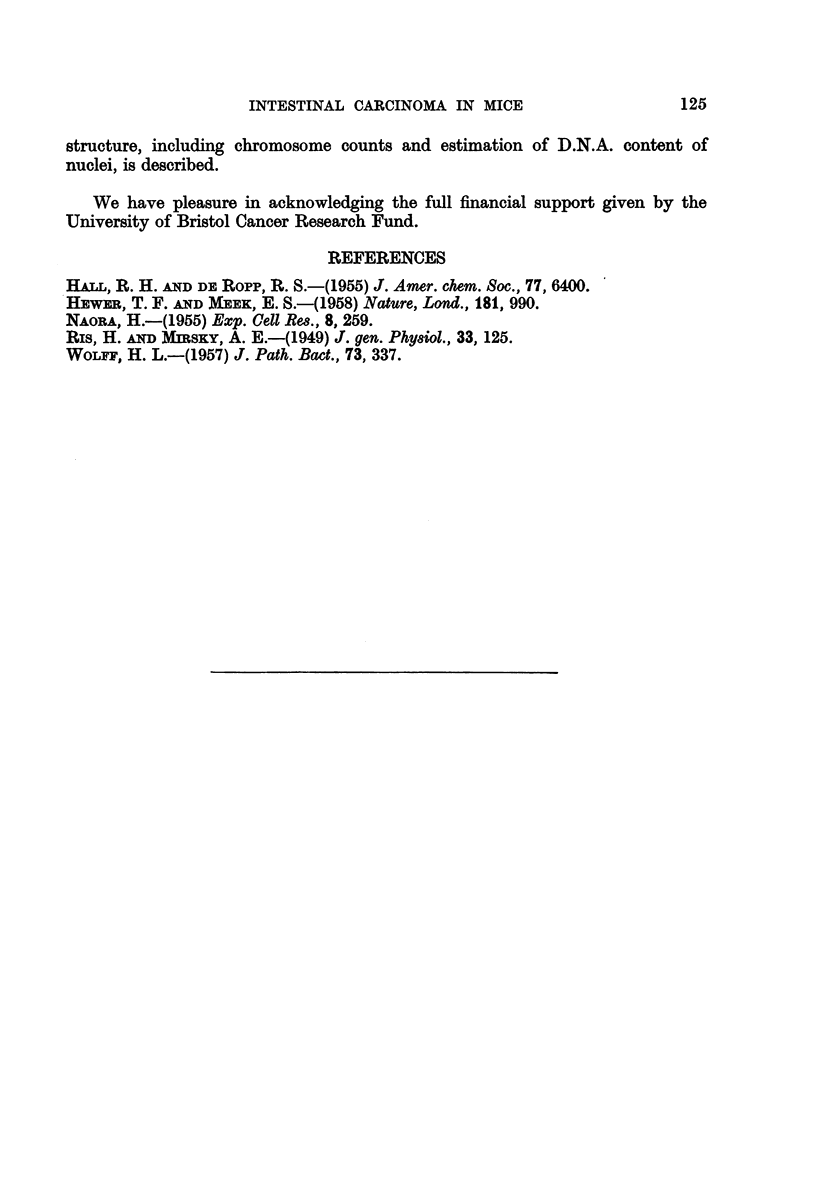# An Intestinal Carcinoma in Mice Following Injection of Herring-Sperm Deoxyribonucleic Acid

**DOI:** 10.1038/bjc.1959.17

**Published:** 1959-03

**Authors:** E. S. Meek, T. F. Hewer

## Abstract

**Images:**


### AN INTESTINAL CARCINOMA IN MICE FOLLOWING INJECTION OF HERRING-SPERM DEOXYRIBONUCLEIC ACID

E. S. MEEK AND T. F. HEWER
*From the Department of Pathology, University of Bristol*

Received for publication December 18, 1958

As a preliminary to an attempt to incorporate in a living animal the deoxyribonucleic acid (D.N.A.) from a malignant tumour, it was thought advisable to perform a number of control experiments including one with a preparation of D.N.A. having no relation to any tumour, so as to exclude a non-specific effect. For this purpose a commercial product derived from herring-sperm was employed (Hewer and Meek, 1958). This was a depolymerised sample extracted by a hot alkaline method.

### METHOD

A single litter of four female line-bred $C_3H$ mice*, three weeks old at the start of the experiment, was used. Two of the mice were injected subcutaneously daily for five days with 0·1 ml. of 5 per cent D.N.A. solution. The other two were used as controls and received no injections at all.

### RESULTS

On the 23rd day of the experiment one of those receiving injections was found dead and the other was moribund. The latter was killed immediately and selected tissues were fixed in Bouin's Fluid. The two other litter mates remained healthy and have developed since into normal adult mice subsequently killed at the age of one year. In both mice that had received injections there was a copious blood-stained peritoneal exudate and a firm pale grey ulcerating carcinoma, about 1 cm. in diameter, arising in the mucosa of the third part of the duodenum. The histological structure of these two tumours was the same, but since there had been some post-mortem autolysis of the mouse that was found dead a detailed study was made only of the other.

Both tumours had penetrated the gut wall and spread over the serous surface, and had also invaded the pancreas. There were some tumour cells free in the peritoneal fluid together with a few histiocytes and lymphocytes ; in fact the tumour had already become established as an ascites-cell tumour, with more than 70 per cent tumour cells in the exudate.

### MORBID ANATOMY

Since the lesions in the two mice were identical but the one found dead had suffered some post-mortem autolysis the following detailed description is based upon the findings in the other.

* The original breeding pairs used to establish this colony were kindly supplied by Dr. J. Craigie, Imperial Cancer Research Fund, Central Laboratories, Mill Hill, London.

The primary tumour arose in the mucosa of the third part part of the duodenum on the mesenteric side but had extended almost completely around the wall without, however, causing any obstruction. There was a small central area of ulceration of the mucosa and the main tumour measured approximately 8 mm. in diameter ; it had invaded the mesentery and had extended into the body of the pancreas without causing any biliary obstruction. On section the tumour was rather friable, of uniform consistency with a few small yellowish necrotic foci, and involved the mucosa and the adjacent pancreatic tissue to such an extent that it was not possible in the gross to determine the exact site of its origin. Some loops of small and large intestine were loosely adherent to the outside of the tumour mass where some small nodules of tumour which had invaded the serous coat had given rise to the peritoneal spread. The mesenteric and para-aortic lymph nodes contained obvious metastases. (In the other mouse there was in addition a metastasis in one of the inguinal lymph nodes). These lymphatic metastases had penetrated the capsule of the lymph nodes and spread into the surrounding tissues to some extent. No metastases were found in the liver or in any extra-abdominal site.

Microscopically, the tumour proved to be a poorly differentiated adenocarcinoma arising from the deeper parts of the mucosal crypts. It was extremely cellular with finely granular cytoplasm and indistinct cell outlines. Most of the tumour was quite anaplastic but fortunately in some areas (Fig. 1) a transition was clearly perceptible between the normal duodenal mucosal crypts and those that had become carcinomatous. Mitoses were numerous and the cells varied considerably in size. The nuclei were vesicular with prominent nucleoli (Fig. 2). In the places where the pancreas was invaded sheets of tumour cells surrounded the pancreatic acini, the cells of which were mostly smaller than those of the tumour and generally distinguishable from them. In the lymph nodes the tumour cells gave no more than a suggestion of acinar arrangement.

Methanol-fixed smears of the ascites cells were stained by the May-Grünwald-Giemsa method and others fixed in buffered neutral formol-saline were stained with Oil Red O. The cells were mostly spherical and varied between 20 and 30 $\mu$. in diameter. Fat droplets were present in the cytoplasm of almost all the cells and a very few showed cytoplasmic budding. The cytoplasm tended to be basophilic. Numerous bizarre nuclear forms were encountered including a few that were binucleate ; in one of the latter (Fig. 3) there was a small nuclear protrusion present. Occasional giant cells were seen.

### TRANSMISSION OF THE TUMOUR

Attempts were made to pass the solid tumour into other $C_3H$ mice subcutaneously ; these were successful in only three of six mice, and in these three subsequent passages could not be achieved. On the other hand the ascites fluid containing the ascites cell form, produced a vigorous growth of the tumour on

---

### EXPLANATION OF PLATE

Fig. 1.—Primary tumour showing transition between normal and neoplastic tissue. Haemotoxylin and eosin. × 195.

Fig. 2.—Primary tumour showing cellular detail. Haemotoxylin and eosin. × 390.

Fig. 3.—Ascites tumour cells including a giant form. May-Grünwald-Giemsa. × 650.

1

2

3

Meek and Hewer.

intra-peritoneal injection with both $C_3H$ and an inbred laboratory strain of albino mice, and has been maintained over the past year.

### CYTOLOGICAL INVESTIGATIONS

Since the purpose of this experiment was to see whether the genetic structure of the cells could be altered by administration of D.N.A., studies were made of the chromosome and D.N.A. content of the tumour. This was more conveniently achieved with the ascites cells than with the solid tumour.

Chromosome counts were made on tumour cells arrested in metaphase by the administration of colchicine (6 $\mu$g. per 10 g. body weight) intraperitoneally to mice already bearing the ascites cell tumour. The cells were harvested at 10 hours after the administration of colchicine and were fixed immediately in aceto-orcein solution. Squash preparations were made and the chromosomes counted with the aid of a simple ocular grid. Most of the cells proved to be triploid, the count in individual cells varying between 56 and 63. In addition to this main group there was a small number of cells with a chromosome number lying between 120 and 130. Two cells were also found with a tetraploid figure of 80 and one cell with approximately 180 chromosomes.

D.N.A. estimations were carried out using a slight modification of the method and apparatus of Wolff (1957) to measure the intensity of Feulgen staining. A 108-watt tungsten ribbon filament lamp was used as the light source for projection of selected cells onto a screen fitted with a photo-electric cell behind a small aperture. Amongst the tumour cells there were lymphocytes present in the peritoneal exudate which could be distinguished from the tumour cells in Feulgen preparations. Since lymphocytes are normal somatic cells, and are therefore presumably diploid, measurement of their D.N.A. content would give normal values as a standard for comparison with the tumour. This amounts to a convenient built-in standard to minimise errors due to variations in staining intensity.

The results show (Fig. 4) that there is very little scatter of D.N.A. values in the normal lymphocyte nuclei whereas those of the tumour show in the main greatly increased values for D.N.A. content and a very much wider scatter, ranging from a few at normal level to a single reading of as much as about ten times the normal. It will be seen from Fig. 4 that the majority of the D.N.A. values lie in the range of twice to four times that of the normal cells.

The sources of error in this method for gauging D.N.A. content have been studied (Ris and Mirsky, 1949; Naora, 1955). It is recognised that there is difficulty in reproducing exactly the intensity of staining in any two preparations; this and other difficulties are increased by the lack of nuclear homogeneity. We were encouraged by the small degree of scatter in the values obtained for normal lymphocyte nuclei to feel that the use of these naturally occurring standards in our preparations did much to meet these objections.

### DISCUSSION

Although we have as yet been unable to initiate any more tumours in this way our findings are of sufficient potential importance to warrant serious consideration. Firstly, we cannot find any report of the natural occurrence of an adenocarcinoma of the duodenum in $C_3H$ mice; secondly, a triploid tumour is not common and, thirdly, its appearance in both the mice injected wlth D.N.A. suggests interference with nucleic acid metabolism.

The original sample of D.N.A. used was small and no more of that batch was available. Another sample was used for later experiments and gave negative results. It is possible that these were not chemically identical and that the first contained some component lacking in the second. Hall and de Ropp (1955),

FIG. 4.—The D.N.A. values of 100 lymphocytes compared with those of 100 tumour cells.

showed that among the breakdown products of D.N.A. that may appear on storage or after autoclaving is kinetin (furfurylaminopurine) which, they concluded, might be formed by the interaction of adenine and 2-deoxy-D-ribose. It is possible that a product of this type had been formed in the sample of herring-sperm D.N.A. and become incorporated, on injection, in the D.N.A. of cells in the mucosa of the gut which were dividing very rapidly in these young mice.

### SUMMARY

Duodenal adenocarcinoma appeared in two young mice given repeated injections of herring-sperm D.N.A. The tumours appeared identical. The histological structure, including chromosome counts and estimation of D.N.A. content of nuclei, is described.

We have pleasure in acknowledging the full financial support given by the University of Bristol Cancer Research Fund.

### REFERENCES

Hall, R. H. and de Ropp, R. S.—(1955) *J. Amer. chem. Soc.*, **77**, 6400.
Hewer, T. F. and Meek, E. S.—(1958) *Nature, Lond.*, **181**, 990.
Naora, H.—(1955) *Exp. Cell Res.*, **8**, 259.
Ris, H. and Mirsky, A. E.—(1949) *J. gen. Physiol.*, **33**, 125.
Wolff, H. L.—(1957) *J. Path. Bact.*, **73**, 337.